# Pairwise association of key lifestyle factors and risk of colorectal cancer: a prospective pooled multicohort study

**DOI:** 10.1002/cnr2.1612

**Published:** 2022-03-03

**Authors:** Eira Roos, Karri Seppä, Olli Pietiläinen, Heidi Ryynänen, Sanna Heikkinen, Johan G. Eriksson, Tommi Härkänen, Pekka Jousilahti, Paul Knekt, Seppo Koskinen, Maarit Laaksonen, Satu Männistö, Teemu Roos, Ossi Rahkonen, Nea Malila, Janne Pitkäniemi, The METCA Study Group

**Affiliations:** ^1^ Department of Public Health University of Helsinki Helsinki Finland; ^2^ Finnish Cancer Registry Institute for Statistical and Epidemiological Cancer Research Helsinki Finland; ^3^ The Finnish Institute for Health and Welfare (THL) Helsinki Finland; ^4^ School of Mathematics and Statistics University of New South Wales Sydney Australia; ^5^ Department of Computer Science University of Helsinki Helsinki Finland; ^6^ School of Health Sciences University of Tampere Tampere Finland

**Keywords:** alcohol, cohort study, colorectal cancer, obesity, physical inactivity, smoking

## Abstract

**Background:**

Several lifestyle factors are associated with an increased risk of colorectal cancer (CRC). Although lifestyle factors co‐occur, in most previous studies these factors have been studied focusing upon a single risk factor or assuming independent effects between risk factors.

**Aim:**

To examine the pairwise effects and interactions of smoking, alcohol consumption, physical inactivity, and body mass index (BMI) with risk of subsequent colorectal cancer (CRC).

**Methods and results:**

We used METCA cohort data (pooled data from seven population‐based Finnish health behavior survey studies during years 1972–2015) consisting of 171 063 women and men. Participants' smoking, alcohol consumption, physical inactivity and BMI measures were gathered, and participants were categorized into those exposed and those not exposed. The incidence of CRC was modeled by Poisson regression with main and interaction effects of key lifestyle factors.

The cohort members were followed‐up through register linkage to the Finnish Cancer Registry for first primary CRC case until the end of 2015. Follow‐up time was 1715, 690 person years.

The highest pairwise CRC risk was among male smokers who had overweight (BMI ≥ 25 kg/m^2^) (HR 1.75, 95% CI 1.36–2.26) and women who had overweight and consumed alcohol (HR 1.45, 95% CI 1.14–1.85). Overall, among men the association of lifestyle factors and CRC risk was stronger than among women. In men, both having overweight and being a smoker combined with any other adverse lifestyle factor increased CRC risk. Among women, elevated CRC risks were observed for those who were physically inactive and who consumed alcohol or had overweight. No statistically significant interactions were detected between pairs of lifestyle factors.

**Conclusions:**

This study strengthens the evidence of overweight, smoking, and alcohol consumption as CRC risk factors. Substantial protective benefits in CRC risk can be achieved by preventing smoking, maintaining BMI to <25 kg/m^2^ and not consuming alcohol.

## 1 INTRODUCTION

Colorectal cancers (CRC) are the third most common site for new cancers and second most common cause of cancer deaths in the world.^1^, ^2^ Although the overall growth rate in CRC incidence has somewhat slowed^3^ the incidence and mortality still increase even in many high‐income countries^4^ and this global trend is also observed in Finland.^5^, ^6^

There are several reasons behind the increasing CRC incidence. Increasing age is the single most important risk factor for CRC, and the increasing life expectancy will increase the number of colorectal cancers in the future.^7^ Males have a higher incidence of colorectal cancer than females.^7^ Several adverse lifestyle factors are associated with an increased risk of CRC.^8^, ^9^, ^10^, ^11^, ^12^ There is convincing evidence on alcohol consumption, with risk for CRC increasing noticeably with heavy drinking.^9^, ^10^ Obesity, especially abdominal obesity, increases the risk^11^ whereas those who are physically active have lower risk.^12^ There is sufficient evidence in humans that tobacco smoking causes colorectal cancer.^13^ Studies have suggested beneficial and protective effects of diets rich in, for example, fruits, vegetables, fish, fiber, and whole grain, while processed and red meat are associated with increased CRC risk.^9^ Family history of CRC increases the risk. Despite twofold risk for CRC for those with first‐degree relatives with CRC, the vast majority of CRC cancers are sporadic, with only <5% of CRC's being related to known genetic mutations (e.g., FAP or HNPCC).^14^

Frequently the influence of lifestyle factors in CRC have been studied focusing upon a single risk factor or assuming independent effects between risk factors in statistical modeling, without properly exploring possible combined effects of risk factors and their interactions. However, in the EPIC‐cohort study with more than 300 000 subjects from nine European countries, two healthy lifestyle factors combined reduced the CRC risk by 13% on average, compared to persons with none of the five studied lifestyle factors (alcohol, physical activity, diet, smoking, and overweight/obesity).^15^ None of the pairwise healthy lifestyle factor effects were significant. The combined effects of lifestyle factors have been studied in large studies in the United States^16^, ^17^ and in Denmark,^18^ reporting higher CRC risk with increasing number of risk factors and larger risk in men than women. Previous studies on interactions between lifestyle factors with regard to CRC risk are few. A recent Canadian study found an additive effect between alcohol consumption and smoking^19^ and the same finding was reported in a smaller South‐Korean study.^20^ A pooled study consisting of five cohort studies and three population‐based case–control studies^21^ found some evidence on interaction between body weight and smoking in relation to CRC, but this finding has not been confirmed.

The aim of this study was to examine the pairwise effects and interaction of smoking, alcohol consumption, physical inactivity and body mass index (BMI) with the risk of primary CRC in a prospective cohort setting.

## 2 MATERIAL AND METHODS

We used data pooled for the METCA consortium (Prospective METa Cohort Study of Cancer Burden in Finland).^22^ The study covers the following survey studies monitoring health behavior between 1972 and 2015: The National FINRISK Study conducted at 5‐year intervals since 1972 (FINRISK),^23^ The Adult Health, Wellbeing, and Services Studies 1 from 2010 to 2011 (ATH1) and 2 from 2012 to 2015 (ATH2),^24^ The Health 2000 Survey (H2000),^25^ The Finnish Mobile Clinic Health Survey from 1972 to 1977 (FMCF),^26^ The Mini‐Finland Health Survey from 1978 to 1980 (MFH),^26^ the Helsinki Health Study from 2000 to 2002 (HHS)^27^ and The Helsinki Birth Cohort Study (HBCS)^28^ (Appendix S1 in Data S1). Exposure assessment includes both survey data and health examinations.

Here, the largest individual study cohorts are ATH1 and ATH2 (*n* = 77 241, see Appendix S1 in Data S1) and FINRISK (*n* = 52 661), but longest follow‐up comes from the FMCF (390 884 person‐years), MFH (142 183), and FINRISK (839 400).

Smoking, alcohol consumption, physical inactivity, and BMI measures were harmonized between the study cohorts and categorized into those exposed and those not exposed. Smoking was grouped into never‐smokers (reference, not exposed) and smokers (ex‐ and current smokers). Regarding alcohol use, subjects reporting using 0 g of alcohol per week (MFH, HHS), per month (FMCF), never use of alcohol (FINRISK, H2000, HBCS), or not using alcohol within the past year (ATH) were categorized as non‐exposed. Accordingly, subjects reporting any, ever, or current use of alcohol were considered as exposed. Applicable information on alcohol use was not available in the FINRISK 1982 survey. Physically inactive (no leisure time physical activity) were categorized into exposed and physically active into not exposed (reference, those with any leisure time activity). Body mass index was divided into those with BMI < 25 kg/m^2^ (reference; not exposed) and having overweight (BMI ≥ 25 kg/m^2^, exposed). Missing item values of a covariate were handled as a separate category in the analysis.

The cohort members were followed‐up through individual register linkage with personal identity codes to the nation‐wide population‐based Finnish Cancer Registry for cancers and to Statistics Finland or the Population Register Centre for deaths.^29^, ^30^ The follow‐up started either from the date of baseline survey or the date when the person turns 50 years, which ever occurred latest. The follow‐up continued until the end of 2013 or 2015 (depending on the cohort), death or emigration.^22^

In total, 1660 incident primary CRC cancers among 171 063 persons during 1715 690 person‐years were observed (Table 1). Approximately one‐third of men (37%) and 44% of women had BMI < 25 kg/m^2^ (Table 2). Of men, 16% reported never consuming alcohol, while nearly a third of the women (34%) reported the same. Around 70% of both men (74%) and women (70%) were active during leisure time, and around one third of men (31%) and two thirds of women (65%) were never‐smokers.

**TABLE 1:** Summary statistics of study cohort characteristics

Population characteristics	Total	Men	Women
Years of baseline of harmonized cohort	1972–2015		
Number of subjects in harmonized cohort	171 063	76 762	94 301
Person years	1 715 690	754 439	961 251
First primary CRC	1660	859	801
Follow‐up years (median [SD])	6 (10)	6 (10)	6 (10)
Age at baseline in years (mean [SD])	57 (16)	56 (15)	58 (16)
Proportion of men/women (%)	45/55		

**TABLE 2:** Prevalence of risk factors of study cohort

		Total		Men		Women	
Risk factors		*N*	%	*N*	%	*N*	%
Smoking	No	83 469	50	23 609	31	59 860	65
	Yes	83 591	50	51 512	69	32 079	35
Alcohol	No	43 256	26	12 227	16	31 029	34
	Yes	122 580	74	62 703	84	59 877	66
Weight (kg)	Normal weight (<25 kg/m^2^)	67 571	41	27 630	37	39 941	44
	Overweight (> = 25 kg/m^2^)	97 929	59	46 948	63	50 981	56
Physical inactivity	Active during leisure time	119 246	72	55 206	74	64 040	70
	Inactive during leisure time	47 011	28	19 494	26	27 517	30

For each pair of lifestyle factors we calculated the sum of person years, number of first primary CRC, and age‐standardized incidence rate. The age standardization was performed with direct standardization using the age distribution of the world 1966 population. The hazard ratios (HRs) of lifestyle factors for CRC were estimated using Poisson regression models based on multiplicative hazard functions. Let $C_{\text{apse}}$ be the number of cancer cases among persons in age group *a*,calendar period p (5‐year periods) and survey study s with values $e=\left(k,l,m,n\right)$ of the four lifestyle factors described by the Poisson distribution $C_{\text{apse}}\;\text{Poisson}\left(\lambda_{\text{apse}}y_{\text{apse}}\right)$ where $\lambda_{\text{apse}}$ is the cancer incidence rate and $y_{\text{apse}}$ is the number of persons years in the stratum. In the first model, we included only the main effects of the lifestyle factors:

(1) $$\log\left(\lambda_{\text{apse}}\right)=\alpha_{\mathrm{aps}}+\beta_{k}^{1}+\beta_{l}^{2}+\beta_{m}^{3}+\beta_{n}^{4}$$

where $\exp\left(\alpha_{\mathrm{aps}}\right)$ is the baseline hazard and $\exp\left(\beta_{k}^{i}\right)$ is the multiplicative main effect of factor i with value k. The baseline hazard was stratified by age (5‐year groups of attained age) and calendar time (5‐year periods) in order to account for variation in the hazard by age and period, and variation in the baseline hazard between studies was modeled by multiplicative study‐specific effects: $\alpha_{\mathrm{aps}}=\alpha_{\mathrm{ap}}+\delta_{s}$. In model M1, we assumed the main effects model for the lifestyle factors, that is, the HR of two factors was the product of the HRs of each lifestyle factor, and made the common statistical assumption of proportional hazards, that is, the HRs were constant in time. In an alternative model, M2, the interaction term $\gamma^{\mathrm{ij}}$ of each pair $\left(i,j\right)$ of lifestyle factors, excluding the interaction terms where either factor was missing, was added to model M1:

(2) $$\log\left(\lambda_{\text{apse}}\right)=\alpha_{\mathrm{aps}}+\beta_{k}^{1}+\beta_{l}^{2}+\beta_{m}^{3}+\beta_{n}^{4}+\gamma^{\mathrm{ij}}$$

Models M1 and M2 were fitted separately for men and women.

Hazard ratios (HR) of main effects $\exp\left(\beta_{k}^{i}\right)$ and $\exp\left(\beta_{l}^{j}\right)$, pairwise effect $\exp\left(\beta_{k}^{i}+\beta_{l}^{j}+\gamma^{\mathrm{ij}}\right)$ and multiplicative interaction $\exp\left(\gamma^{\mathrm{ij}}\right)$ are reported with their 95% confidence intervals (CI). In order to test interaction for pairs of lifestyle factors, we compared the fit of models M1 and M2 by using the likelihood ratio test. Heterogeneity between men and women in (i) the effect of each lifestyle factor and (ii) the effects of each pair of lifestyle factors was evaluated by analyzing men and women combined. By using the likelihood ratio test, we compared models where the corresponding effects were assumed to be either sex‐specific or not, and the baseline hazard and the effects of the other lifestyle factors were stratified by sex. *p*‐Values were adjusted for multiple testing using the method of Benjamini and Hochberg.^31^

The study was approved by the Finnish Institute for Health and Welfare (Permit no. THL/1091/6.02.00/2015 and THL/679/6.02.00/2018).

## 3 RESULTS

Men and women who had overweight had an increased CRC risk (HR 1.24, 95% CI 1.07–1.44 and HR 1.20, 95% CI 1.03–1.39, respectively) when adjusted for age, calendar time, study cohort, and other lifestyle factors (Table 3). Smoking increased CRC risk in men (HR 1.38, 95 CI 1.18–1.63), but not in women (HR 0.98, 95% CI 0.82–1.16). None of the other studied factors were significantly related to CRC risk in men or in women.

**TABLE 3:** Adjusted hazard ratios (HR) and 95% confidence intervals (CI) of colorectal cancer lifestyle risk factors

	Men	CI		Women	CI	
	HR^a^	2.5%	97.5%	HR^a^	2.5%	97.5%
Use alcohol vs. no use of alcohol	1.16	0.95	1.41	1.13	0.96	1.33
Overweight or obese vs. normal weight	1.24	1.07	1.44	1.20	1.03	1.39
Ex‐ or current smoker vs. never smoker	1.38	1.18	1.63	0.98	0.82	1.16
No leisure time exercise vs. any leisure time exercise	1.06	0.91	1.24	1.13	0.97	1.31

^a^ Adjusted for study, age, calendar time, and other lifestyle risk factors.

**FIGURE 1:**
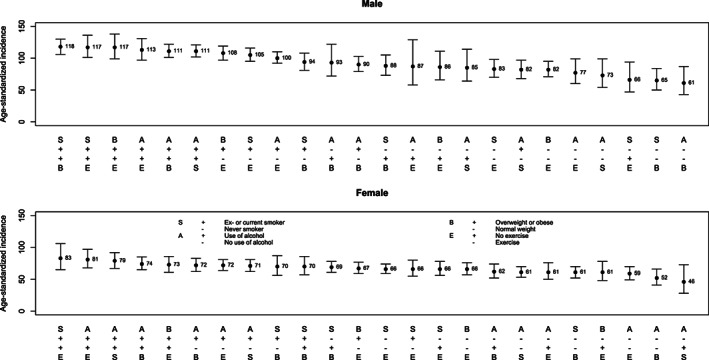
Age‐standardized CRC incidence rate and 95% confidence intervals for pairwise lifestyle factor pairs by sex

The ordered age‐standardized incidence rates for all lifestyle factor pairs are plotted in Figure 1 by gender. CRC incidence was in general higher in men (ranging from 118 to 61 per 100 000) than in women (ranging from 83 to 46 per 100 000). Men who smoked or had smoked and had overweight had the highest age standardized CRC rate (118 per 100 000, 95% 106–130, Figure 1). In women, the highest age standardized CRC rates were observed in those who smoked and were physical inactive (83 per 100 000, 95% CI 65–106). Women who used alcohol and had overweight had an adjusted incidence rate of 74 per 100 000 (95%CI 65–85).

The number of CRCs, person‐years and adjusted CRC HR for all lifestyle factor pairs are presented in Table 4 for men and women separately. Men exposed to any two of the four studied lifestyle factors (smoking, use of alcohol, physical inactivity, or having overweight) had significantly elevated HRs compared to men not exposed to these factor pairs. The only exception was in the use of alcohol and physical inactivity, where the pairwise HR was not significantly elevated. Men who smoked or had smoked and had overweight had the highest CRC risk (HR 1.75, 95% CI 1.36–2.26) compared to male never‐smoked with BMI < 25. Women who used alcohol and had overweight had an elevated CRC risk (HR 1.45, 95% CI 1.14–1.85) compared to women with normal weight who did not use alcohol. Women who were physically inactive and consumed alcohol (HR 1.29, 95% CI 1.02–1.63) or had overweight (HR 1.36, 95% CI 1.10–1.67) had significantly elevated CRC risks. Male smokers who had overweight, used alcohol, or were physically inactive had higher HR of CRC than women with similar lifestyle factor pairs (*p* = .03).

**TABLE 4:** Number of colorectal cancers and person‐years, age standardized incidence and adjusted hazard ratios (HR) of colorectal cancer for lifestyle risk factor pairs by sex

		Men			Women		
Risk factor pair		Cancers/person‐years	Age standardized incidence	HR (95% CI)	Cancers/person‐years	Age standardized incidence	HR (95% CI)
BMI	Alcohol						
Normal	No	44/50 228	61 (43, 87)	1.00	96/142 109	52 (41, 66)	1.00
Normal	Yes	226/239 678	90 (79, 103)	1.24 (0.91, 1.69)	191/270 185	72 (62, 83)	1.37 (1.07, 1.75)
Overweight	No	81/62 938	93 (72, 122)	1.35 (0.96, 1.92)	243/235 737	62 (52, 74)	1.43 (1.13, 1.81)
Overweight	Yes	479/379 666	111 (101, 122)	1.51 (1.11, 2.04)	241/279 610	74 (65, 85)	1.45 (1.14, 1.85)
Interaction				0.90 (0.62, 1.31)			0.74 (0.55, 0.99)
P‐interaction^a^				0.695			0.263
Smoking	Alcohol						
Never smoker	No	58/57 148	73 (54, 99)	1.00	320/339 945	61 (53, 70)	1.00
Never smoker	Yes	144/154 866	82 (68, 97)	1.11 (0.82, 1.50)	264/324 267	71 (62, 81)	1.05 (0.88, 1.26)
Ex‐ or current smoker	No	68/56 674	85 (64, 114)	1.31 (0.93, 1.83)	25/43 590	46 (28, 73)	0.66 (0.44, 0.99)
Ex‐ or current smoker	Yes	570/472 592	111 (102, 121)	1.56 (1.18, 2.05)	172/229 540	79 (67, 92)	1.14 (0.93, 1.40)
Interaction				1.08 (0.74, 1.58)			1.64 (1.04, 2.57)
P‐interaction^a^				0.764			0.263
Exercise	Alcohol						
Exercises	No	89/78 969	77 (60, 99)	1.00	194/225 218	59 (49, 70)	1.00
Exercises	Yes	525/470 693	100 (92, 110)	1.08 (0.86, 1.36)	307/409 865	72 (64, 81)	1.09 (0.90, 1.33)
No exercise	No	36/33 698	87 (58, 129)	0.88 (0.60, 1.28)	150/154 296	61 (50, 76)	1.08 (0.87, 1.33)
No exercise	Yes	186/153 177	113 (97, 131)	1.20 (0.92, 1.55)	132/142 949	81 (68, 97)	1.29 (1.02, 1.63)
Interaction				1.25 (0.83, 1.89)			1.10 (0.82, 1.47)
P‐interaction^a^				0.555			0.695
BMI	Smoking						
Normal	Never smoker	65/86 486	65 (50, 84)	1.00	194/275 640	61 (52, 70)	1.00
Normal	Ex‐ or current smoker	205/203 335	94 (81, 108)	1.43 (1.09, 1.87)	92/137 165	70 (56, 87)	1.11 (0.87, 1.42)
Overweight	Never smoker	132/124 317	88 (73, 105)	1.29 (0.97, 1.71)	384/384 107	69 (61, 78)	1.27 (1.07, 1.52)
Overweight	Ex‐ or current smoker	429/319 072	118 (106, 130)	1.75 (1.36, 2.26)	100/132 922	70 (57, 86)	1.12 (0.87, 1.43)
Interaction				0.95 (0.69, 1.32)			0.79 (0.57, 1.09)
P‐interaction^a^				0.767			0.555
Exercise	Smoking						
Exercises	Never smoker	161/164 629	83 (70, 98)	1.00	378/443 092	66 (59, 74)	1.00
Exercises	Ex‐ or current smoker	456/384 939	105 (95, 116)	1.32 (1.10, 1.58)	124/192 807	66 (55, 80)	0.90 (0.73, 1.12)
No exercise	Never smoker	40/46 441	66 (47, 94)	0.90 (0.64, 1.26)	207/218 689	66 (56, 78)	1.07 (0.90, 1.27)
No exercise	Ex‐ or current smoker	181/140 984	117 (101, 136)	1.46 (1.18, 1.82)	72/79 913	83 (65, 106)	1.21 (0.93, 1.56)
Interaction				1.23 (0.85, 1.80)			1.25 (0.89, 1.75)
P‐interaction^a^				0.555			0.555
Exercise	BMI						
Exercises	Normal	201/219 487	82 (71, 95)	1.00	213/303 236	66 (57, 76)	1.00
Exercises	Overweight or obese	408/325 851	108 (97, 119)	1.21 (1.02, 1.43)	289/329 123	67 (59, 77)	1.15 (0.96, 1.37)
No exercise	Normal	65/69 565	86 (66, 111)	0.99 (0.75, 1.29)	76/109 450	61 (48, 78)	1.04 (0.81, 1.33)
No exercise	Overweight or obese	155/114 727	117 (99, 138)	1.33 (1.08, 1.63)	195/184 515	73 (61, 86)	1.36 (1.10, 1.67)
Interaction				1.11 (0.80, 1.55)			1.14 (0.84, 1.55)
P‐interaction^a^				0.695			0.689

^a^ P‐interaction: *p*‐value for H_0_: HR (interaction) = 1.00, corrected for multiple comparisons (Benjamini–Hochberg).

Measures of interactions on a multiplicative scale between pairwise lifestyle factors are shown in Table 4 separately for men and women. In men no statistically significant interactions were detected. In women, a positive interaction between smoking and alcohol consumption was found: interaction on the multiplicative scale 1.64 (95% CI 1.04–2.57). The estimated joint effect on the HR scale of smoking and alcohol together was greater (64%) than the product of the estimated effects of smoking and alcohol alone, so that there was positive interaction on the multiplicative scale.^32^ A negative interaction (HR 0.74, 95% CI 0.55–0.99) was detected in women between alcohol use and having overweight. This implies that the joint effect of these factors was smaller than the product of these two effects alone. However, after correcting for multiple comparisons, none of the interactions remained statistically significant between pairs of lifestyle factors.

When excluding the cohorts with shortest follow‐ups (ATH1 and ATH2) the main effect of alcohol in men changed from HR 1.16 to HR 1.36 and it became statistically significant. The effects of all other lifestyle factors did not change substantially.

## 4 DISCUSSION

We found several significantly elevated CRC risks of pairwise combinations of major lifestyle factors, especially in males. The highest risk was among male smokers who had overweight. In women, the highest risk was among those who consumed alcohol and in addition had overweight. Moreover, among men both having overweight and smoking combined with any other studied adverse lifestyle factor increased the risk. In women, an elevated risk was found among physically inactive women who consumed alcohol or had overweight.

In our study, smoking was associated with CRC only among men. This may be due to the long carcinogenic pathway requiring decades of exposure to tobacco smoke to result in CRC.^33^ Follow‐up started in the 1970s, when smoking was less common among women than in later years. It may be that the women in our cohort have not been exposed to tobacco smoke long enough for CRC to develop during follow‐up. Our results on overweight are in line with previous studies.^11^, ^34^ After exclusion of cohorts with short follow‐ups our findings on alcohol are in line with previous studies.^35^ Physical activity has been shown to reduce the risk of colon cancer^12^ while the evidence is less convincing for rectal cancers.^36^ Our findings support this as we see a CRC risk reduction that does not reach statistical significance.

We found several lifestyle factor pairs to be significantly associated with an increased CRC risk when compared to individuals with neither of the factors. The previous EPIC study did not find any of the two lifestyle factors being associated with reduced CRC risk, when compared to individuals with no or only one healthy lifestyle factor.^15^ In the EPIC study, significant protective effects were observed only with three or more healthy lifestyle factors combined. Comparing our results with EPIC is not straight forward as the reference groups were different and our study did not have information on diet. The reference group in the EPIC study consisted of individuals with no healthy lifestyle factors, while in our study the individuals in the reference group could have unfavorable or favorable factors except for those included in studied lifestyle factor pair. Furthermore, we focused on pairwise effects of lifestyle factors, instead of exploring the effects with more than two factors combined. In our study, the follow‐up periods start between 1970 and 2015, while in the EPIC study follow‐up started between 1992 and 2000, allowing us a much longer incubation period.

We detected no statistically significant interactions between pairs of lifestyle factors, when adjusted for multiple comparisons. The highest interactive effect was in women, where smoking combined with alcohol consumption resulted in much higher CRC risks than was expected based on their individual effects. Two recent studies have found a synergistic effect between alcohol consumption and smoking regarding CRC risk.^19^, ^20^ It has been proposed that alcohol may act as a solvent for tobacco carcinogens thus making tobacco more toxic.^37^

Studies in the METCA cohort have varying follow‐up times, which could influence the results. We have previously performed sensitivity analyses for time dependence of exposure effects, where we excluded the first 2 years of follow‐up or follow‐up longer than 10 years. Neither detection nor information bias had a notable effect in the reported results.^22^

In 2018, around 1.8 million people were diagnosed with CRC globally^38^ and the prediction is that CRC rates continue to increase with increased economic development. In Finland, the average age‐standardized incidence rate for CRC was 29.7/100 000 in men and 22.2/100 000 in women in 2015–2019^5^ and the incidence has increased on average 0.7% in men 1990–2019 and 1.5% between 2011 and 2019 in women.^5^ The incidence increase has been steepest among men with basic education (from 16.7/100 000 in 1976–1979 to 31.8 in 2010–2014).^6^ The results from our study may be generalizable to western populations.

The strengths of our study include a large sample size, high‐quality exposure data, and reliable cancer information from seven decades. Cancer diagnoses are based on conclusive register data on all diagnosed cancers in Finland.^29^, ^30^ We had a long follow‐up time period and practically no losses to follow‐up. This enables reliable evaluation of exposures with long effect latency. Our cohort studies include key lifestyle factors with validated^6^ measures. The likely influence of dichotomization of key lifestyle factors would be an underestimation of true HRs for CRC.

A limitation of our study was that the data for exposures were mostly self‐reported and gathered in a single time‐point. However, some of the health data were based on face‐to‐face health examination. The self‐reporting may have affected some factors more severely, such as reporting alcohol consumption and weight. During long recruitment time, the prevalence of lifestyle factors has also changed somewhat. Long recruitment and follow‐up time is both an advantage and a disadvantage in this study. Although lifestyle is rather permanent, respondents may have quit smoking, gained weight, increased use of alcohol, and more over time. Also, our measure of physical activity included only leisure time physical activity, thus lacking information on work time activity.

Phrasing of the survey questions varied between different studies, which posed challenges to data harmonization. For example, regarding alcohol consumption the aim was to identify never‐users. For example, the HBCS cohort selected life‐long never‐users of alcohol, while in some other studies, the question on alcohol consumption referred to more recent or current use (**22**). With respect to physical activity, the aim was to measure leisure–time physical activity. Also, here the wording of the question differed somewhat between the studies. In HBCS and HHS, the question was formulated in a way, where persons with even very slight physical activity were categorized as physically active. In addition, HHS is an employee cohort, where also physical activity while commuting (e.g., walking or cycling to work) was considered as leisure–time physical activity. These lead to differences in exposure prevalence between the included cohorts. In BMI, the reference group (normal weight) also included those with underweight. These variations in the definitions of the reference groups may affect the results and potentially attenuate our findings.

This study strengthens the evidence of overweight, smoking, and alcohol consumption as CRC risk factors. Substantial protective benefits in CRC risk can be achieved by preventing smoking, maintaining BMI to <25 kg/m^2^ and not consuming alcohol.

## AUTHOR CONTRIBUTIONS

**Eira Roos:** Conceptualization (equal); investigation (equal); project administration (equal); writing – original draft (equal). **Karri Seppä:** Data curation (equal); formal analysis (equal); methodology (equal); visualization (equal); writing – original draft (equal). **Olli Pietiläinen:** Formal analysis (equal); investigation (equal); methodology (equal); writing – review and editing (equal). **Heidi Ryynänen:** Data curation (equal); methodology (equal); writing – review and editing (equal). **Sanna Heikkinen:** Data curation (equal); investigation (equal); project administration (equal); writing – review and editing (equal). **Johan G. Eriksson:** Validation (equal); writing – review and editing (equal). **Tommi Härkänen:** Validation (equal); writing – review and editing (equal). **Pekka Jousilahti:** Validation (equal); writing – review and editing (equal). **Paul Knekt:** Validation (equal); writing – review and editing (equal). **Seppo Koskinen:** Writing – review and editing (equal). **Maarit Laaksonen:** Writing – review and editing (equal). **Satu Männistö:** Validation (equal); writing – review and editing (equal). **Teemu Roos:** Investigation (equal); methodology (equal); writing – review and editing (equal). **Ossi Rahkonen:** Conceptualization (equal); investigation (equal); methodology (equal); resources (lead); supervision (equal); validation (equal); writing – review and editing (equal). **Nea Malila:** Conceptualization (equal); investigation (equal); supervision (equal); validation (equal); writing – review and editing (equal). **Janne Pitkäniemi:** Conceptualization (equal); methodology (equal); resources (equal); supervision (equal); validation (equal); writing – review and editing (equal).

## CONFLICT OF INTERESTS

The authors have stated explicitly that there are no conflicts of interest in connection with this article. The authors have been independent from funders.

## ETHICS STATEMENT

The study was approved by The Finnish Institute for Health and Welfare (Permits no. THL/1091/6.02.00/2015 and THL/679/6.02.00/2018).

## Supporting information


**Appendix** S1: Supporting information

## Data Availability

Data availability statement Research data are not shared. According to Finnish laws and regulations, individual‐level sensitive data can only be made available for researchers who fulfil legal requirements for access to personal sensitive data.
