# Supplementary material for: Pairwise association of key lifestyle factors and risk of colorectal cancer: a prospective pooled multicohort study
Source: Cancer Rep (Hoboken). 2022 Mar 3;5(11):e1612. doi: 10.1002/cnr2.1612 (PMC9675367; doi:10.1002/cnr2.1612)
Supplement: Supplementary file 1 — Appendix S1: Supporting information [file CNR2-5-e1612-s001.docx]

Supplementary Appendix 1: Basic characteristics and distributions of the risk factors in the study cohorts

| Population characteristics | | ATH1 | | ATH2 | | FMCF | | FINRISK | | H2000 | | HBCS | | HHS | | MFH | |
| --- | --- | --- | --- | --- | --- | --- | --- | --- | --- | --- | --- | --- | --- | --- | --- | --- | --- |
| Years of baseline of harmonized cohort | | 2010-2011 | | 2012-2015 | | 1972-1977 | | 1972-2012 | | 2000-2001 | | 2000 | | 2000-2002 | | 1978-1980 | |
| N of subjects in harmonized cohort | | 12129 | | 65112 | | 19144 | | 52661 | | 6362 | | 1988 | | 6563 | | 7104 | |
| Person years | | 60116 | | 119258 | | 390884 | | 839400 | | 64926 | | 24945 | | 73977 | | 142183 | |
| First primary cancers | | 86 | | 173 | | 370 | | 710 | | 82 | | 35 | | 45 | | 159 | |
| Follow-up years (median (SD)) | | 6 (1) | | 2 (1) | | 20 (10) | | 16 (10) | | 11 (5) | | 13 (2) | | 14 (4) | | 20 (9) | |
| Age at baseline (mean (SD)) | | 66 (12) | | 68 (11) | | 45 (15) | | 48 (12) | | 57 (15) | | 60 (3) | | 49 (7) | | 52 (14) | |
| Proportion of men/women (%) | | 43 / 57 | | 43 / 57 | | 50 / 50 | | 48 / 52 | | 44 / 56 | | 46 / 54 | | 21 / 79 | | 46 / 54 | |
|  |  |  |  |  |  |  |  |  |  |  |  |  |  |  |  |  |  |
| Risk factors | | N | % | N | % | N | % | N | % | N | % | N | % | N | % | N | % |
| Smoking | No | 6014 | 53 | 33012 | 53 | 10296 | 54 | 22807 | 44 | 3326 | 53 | 836 | 42 | 3213 | 51 | 3965 | 56 |
|  | Yes | 5436 | 47 | 29527 | 47 | 8767 | 46 | 29553 | 56 | 2987 | 47 | 1136 | 58 | 3055 | 49 | 3130 | 44 |
| Alcohol | No | 3215 | 28 | 17718 | 29 | 8203 | 43 | 9243 | 18 | 1138 | 20 | 54 | 3 | 434 | 7 | 3251 | 46 |
|  | Yes | 8281 | 72 | 44016 | 71 | 10863 | 57 | 42966 | 82 | 4636 | 80 | 1922 | 97 | 6052 | 93 | 3844 | 54 |
| Weight | Normal weight | 4431 | 38 | 23651 | 38 | 10085 | 53 | 20475 | 40 | 1808 | 35 | 583 | 29 | 3302 | 51 | 3236 | 46 |
|  | Other | 7240 | 62 | 38713 | 62 | 9051 | 47 | 31057 | 60 | 3397 | 65 | 1403 | 71 | 3212 | 49 | 3856 | 54 |
| Physical inactivity | Physically active | 7887 | 70 | 44619 | 71 | 14129 | 74 | 35821 | 69 | 4160 | 70 | 1732 | 87 | 6398 | 98 | 4500 | 63 |
|  | Physically inactive | 3461 | 30 | 18089 | 29 | 4961 | 26 | 15792 | 31 | 1744 | 30 | 251 | 13 | 119 | 2 | 2594 | 37 |

ATH= The Adult Health, Wellbeing, and Services Studies, FMCF=The Finnish Mobile Clinic Health Examination Follow-up Survey, FINRISK=National FINRISK Study, H2000=Health 2000 Survey, HHS=Helsinki Health Study, MFH=Mini-Finland Health Study
